# Aberrant DNA methylation of drug metabolism and transport genes in nodular goiter

**DOI:** 10.1186/1756-6614-4-15

**Published:** 2011-10-12

**Authors:** Lihong Zhang, Jing Shi, Li Xu, Bingyin Shi, Peng Hou, Meiju Ji

**Affiliations:** 1 Department of Endocrinology, The First Affiliated Hospital of Xi'an Jiaotong University School of Medicine, Xi'an 710061, the People's Republic of China

**Keywords:** Nodular goiter, Solute carrier (SLC) family, Cytochrome P450 (CYP) family, ATP binding cassette (ABC) family, Drug metabolism and transport genes, DNA methylation

## Abstract

The genes encoding drug-metabolizing enzymes and transporters play an important role in maintaining the normal life processes of human body. Their disorder or defect will lead to the occurrence and development of various diseases. Currently, most of studies have focused on genetic variations in these genes, however, in the present study, we analyzed promoter methylation of 11 drug metabolism and transport genes in a cohort of nodular goiter and normal thyroid tissues using methylation-specific PCR (MSP). Our data first revealed a distinct methylation profiling in drug metabolism and transport genes between nodular goiter and normal thyroid tissues, particularly *ABCB4*, *CYP1B1 *and *CYP24A1 *and *SLC1A2*. Given these genes contribute to the development and progression of various diseases, such as multidrug resistance and tumorigenesis, these epigenetic events may thus play a critical role in the pathogenesis of nodular goiter.

## Findings

The genes encoding drug-metabolizing enzymes and transporters play an important role in transporting various kinds of molecules import or export the cells, which is closely associated with the development of various human diseases, mainly including solute carrier (SLC) superfamily, cytochrome P450 (CYP) superfamily and ATP binding cassette (ABC) superfamily [1]. To date, most of studies focused on investigating SNPs or gene mutation in these genes, however, it is has recently been reported that epigenetic mechanisms were involved in the regulation of these genes [2]. In the present study, we choose 11 drug metabolism and transport genes, including *ABCB1*, *ABCB4*, *ABCG2*, *CYP1A1*, *CYP1B1*, *CYP24A1*, *CYP27B1*, *CYP39A1*, *SLC1A2*, *SLC19A3*, and *SLC26A2*, to detect their methylation status of promoter region in a cohort of nodular goiter and normal thyroid tissues using methylation-specific PCR (MSP).

Methylation analysis of thyroid tissues was carried out in a series of 27 nodular goiter and 23 normal thyroid paraffin-embedded tissues, which were obtained from the Department of Pathology of the First Affiliated Hospital of Xi'an Jiaotong University School of Medicine. All samples underwent histological examination by a senior pathologist. The genomic DNA was isolated from paraffin-embedded tissues using xylene to remove the paraffin and sodium dodecyl sulfate (SDS) and proteinase K to digest tissues, followed by standard phenol-chloroform extraction and ethanol precipitation of DNA. DNA was subsequently treated with sodium bisulfite to detect the methylation status of these 11 genes using methylation-specific PCR (MSP) as described previously [3]. Normal leukocyte DNA was methylated *in vitro *with Sss I methylase (New England Biolabs, Beverly, MA) to generate completely methylated DNA as a positive control. The primer sequences and their annealing temperatures were presented in Table 1. To examine the role of DNA methylation in the regulation of gene expression, we treated 3 thyroid cancer cell lines, including FTC133, K1, and C643, with 5 μM demethylation agent 5-Aza-2'-dC for 5 days to induce the expression of the methylated genes. SPSS17.0 software was used for data analysis, and data were compared using chi-square test or the *t *test. The risk of gene methylation to the various clinical variates, including age, gender, a family history of thyroid disease (such as Graves' disease, goiter, thyroid adenoma and hashimoto thyroiditis (HT); the members include the patient's immediate families within 3 generations), and the level of Tg and TSH, was analyzed using the logistic regression. *P *values < 0.05 were considered significant.

**Table 1:** Methylation-specific PCR (MSP) primers used in the present study

Genes	Allele	Forward (5'→3')	Reverse (5'→3')	Length (bp)	Annealing temperature (°C)
***ABCB1***	M	CGAGGAATTAGTATTTAGTTAATTCGGGTCGG	ACTCAACCCACGCCCCGACG	95	60

	U	TGAGGAATTAGTATTTAGTTAATTTGGGTTGG	ACTCAACCCACACCCCAACA	95	57

***ABCB4***	M	GGTAAGAGCGGTAGGTTGC	GAAAAACGCCTACCGTTACA	121	59

	U	GGTAAGAGTGGTAGGTTGT	AAAAAACACCTACCATTACA	121	55

***ABCG2***	M	ATTTGTGCGTTAGCGTTTTC	CTCCGAAATCGAACGAAATA	149	59

	U	GTAATTTGTGTGTTAGTGTTTTT	CCTCCAAAATCAAACAAAATAAA	149	57

***CYP1A1***	M	TCGGCGTACGTAAGTTAGTC	AAACACAAAAATCCGACGA	113	59

	U	GTTGGTGTATGTAAGTTAGTT	AAAACACAAAAATCCAACAA	113	56

***CYP1B1***	M	CGCGTTTTTAAGTCGAGC	ACCCACGTTTCCATTATACG	125	58

	U	GGGTGTGTTTTTAAGTTGAGT	ACCCACATTTCCATTATACAATA	125	56

***CYP24A1***	M	ATGTTTTGAGGTTGTCGC	AAAATCGAAACTTAACGATTCT	140	57

	U	TTAATGTTTTGAGGTTGTTGT	AAAATCAAAACTTAACAATTCTAAA	140	55

***CYP27B1***	M	TTAGAGTGTTTTATCGCGTTC	CTCGTATAACCTCGACAACC	164	58

	U	TTTTTAGAGTGTTTTATTGTGTTT	AACTCATATAACCTCAACAACCC	164	55

***CYP39A1***	M	TAATGTAGTTCGTCGGGTTTC	AACCAACGCGAAAAAAATAC	152	59

	U	GGGTAATGTAGTTTGTTGGGTTTT	CAACCAACACAAAAAAAATACAA	152	57

***SLC1A2***	M	AGTTGAAGCGGGTGTTTC	GAAATAAAACGCAAACGACC	110	58

	U	AGTTGAAGTGGGTGTTTT	AAAATAAAACACAAACAACC	110	57

***SLC19A3***	M	GTTTGGACGTTCGGATTC	CGCGACTATCGAATAAATCC	114	57

	U	AAGGTTTGGATGTTTGGATTT	ACCCACAACTATCAAATAAATCC	114	55

***SLC26A2***	M	GAGGTGGTCGATCGTAAAC	CGTAACGTTAACTCCTCCG	139	59

	U	AAAGAGGTGGTTGATTGTAAAT	TCCATAACATTAACTCCTCCAC	139	57

**M**, mehthylation-specific primers; **U**, unmenthylation-specific primers

**Figure 1:**
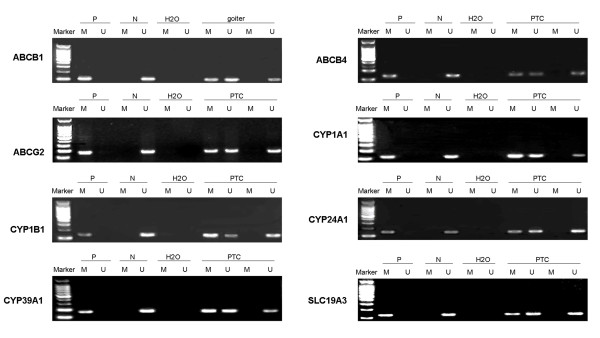
**Representative MSP results of 8 drug metabolism and transport genes in PTC**. *In vitro *methylated DNA was used as positive control for methylated gene (P), bisulfite-modified normal leukocyte DNA as positive control for unmethylated gene (N), and H_2_O as blank control to confirm the specificity of MSP. M, methylated gene; U, unmethylated gene.

**Figure 2:**
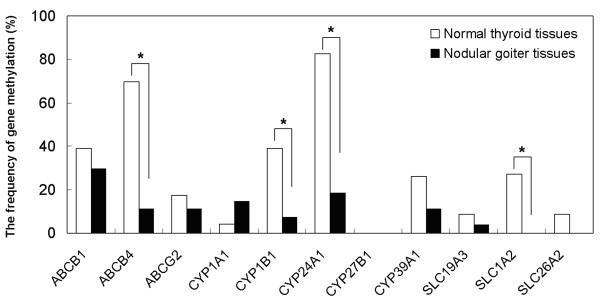
**The methylation frequency of 11 drug metabolism and transport genes in nodular goiter and normal thyroid tissues**. A total of 27 nodular goiter and 23 normal thyroid tissues were analyzed for this study. MSP was used to detect promoter methylation of 11 genes, including *ABCB1*, *ABCB4*, *ABCG2*, *SLC1A2*, *SLC19A3*, *SLC26A2*, *CYP1A1*, *CYP1B1*, *CYP24A1*, *CYP27B1 *and *CYP39A1*. *, *P *< 0.05.

As shown in Figures 1 and 2, 8 of 11 genes were methylated in nodular goiter tissues, ranging from 3.7% to 29.6%. Ten of 11 genes were methylated in normal thyroid tissues, ranging from 4.4% to 82.6%. The methylation rate of these genes, except for *CYP1A1*, was higher in normal thyroid tissues than nodular goiter tissues. Among of them, there was a significantly distinct methylation profiling of *ABCB4*, *CYP1B1 *and *CYP24A1 *and *SLC1A2 *between nodular goiter and normal thyroid tissues (*P *< 0.05) (Figure 2). Promoter methylation of *ABCG2 *was significantly positively associated with a family history of thyroid diseases (*P *< 0.05). The multivariable analyses showed that no significant difference was found between gene methylation and age, gender, a family history of thyroid disease, and the level of Tg and TSH (data not shown).

To date, most of studies have focused on polymorphisms (e.g. SNPs) or gene mutation in drug metabolism and transport genes in some human diseases, including thyroid disease, such as Graves' disease [4]. Unlike the previous studies, this study systematically analyzed promoter methylation of drug metabolism and transport genes in nodular goiter tissues. Promoter methylation is the most common epigenetic events that aberrantly regulate gene expression in different tissues, which play a key role in the early disease [5]. The pathogenesis of nodular goiter is multifactorial and probably differs from patient to patient. In contrast to the endemic goiter, iodine deficiency is not a primary causal factor. Environmental factors, such as natural goitrogens, iodine intake, malnutrition, drugs, stress, pollution or infections, constitutional factors, such as female gender, and several genetic factors, such as circulating thyroid growth factors, contribute to different degree to the development of nodular thyroid enlargement. However, in the present study, our data suggested that aberrant methylation of some genes, such as drug metabolism and transport genes, may play an important role in the pathogenesis of nodular goiter. In support of this, a previous study also showed that the Platelet-derived growth factor B-chain was also abnormally methylated in multinodular goiters [6].

ABCB4 belongs to the multidrug resistance subfamily, encoding a full transporter and member of the p-glycoprotein family of membrane proteins with phosphatidylcholine as its substrate [7], which was more frequently methylated in normal thyroid tissue compared with nodular goiter in the present study. Of note, this gene was highly expressed in various human cancers [8,9]. Its overexpression may induce multidrug resistance and create a waste of energy [7]. CYP1B1 belongs to the cytochrome P450 family 1 and is a major member of the extrahepatic xenobiotic-metabolizing CYP enzyme family. It can transform estrogen to strong carcinogenic compounds 4-hydroxy estrogen, and is overexpressed in various malignant tumors, and promotes the development of tumors [10,11]. *CYP1B1 *was significantly lowly methylated in nodular goiter compared with normal thyroid tissues in the present study, suggesting that its aberrant expression may play a key role in in the pathogenesis of nodular goiter. *CYP24A1 *is relevant with the metabolism of vitamin D3, which emerged as a protective factor for human body. *CYP24A1 *encodes a mitochondrial protein which initiates the degradation of 1,25-dihydroxyvitamin D3, the physiologically active form of vitamin D3, by hydroxylation of the side chain [12]. A previous study showed that the administration of calcitriol in combination with *CYP24A1 *inhibitor enhanced antiproliferative effects, increased systemic calcitriol exposure, and promoted the activation of caspase-independent apoptosis pathway [13]. Interestingly, a very recent study showed that that *CYP24A1 *expression is marked increased in papillary thyroid cancer (PTC) compared with normal thyroid tissues [14]. It is consistent with our recent findings that *CYP24A1 *was significantly lowly methylated in PTC compared with normal tissues (Lihong Zhang, Jing Shi, Meiju Ji, Guanjun Zhang, Jiao Fu, Li Xu, Bingyin Shi, and Peng Hou: Methylation analysis of drug metabolism and transport genes in papillary thyroid cancer, submitted). *SLC1A2 *encodes a membrane-bound protein which is the principal transporter that clears the excitatory neurotransmitter glutamate from the extracellular space at synapses in the central nervous system, to prevent neuronal damage from excessive activation of glutamate receptors [15]. Importantly, our data showed that demethylation agent 5-Aza-2'-dC could induce the expression of these methylated genes in at least one cell line, indicating that expression of these genes could be regulated by promoter methylation (data not shown).

In conclusions, although nodular goiter is a benign disease, it seriously influences the quality of life of many patients because it usually causes dyspnea and swallowing difficulties. Thus, it is amazing if we can inhibit the occurrence or proceeding of this disease. In the present study, we observed that a distinct methylation profiling in drug metabolism and transport genes between nodular goiter and normal thyroid tissues, suggesting that aberrant expression of these genes may play an important role in the pathogenesis of nodular goiter.

## Competing interests

The authors declare that they have no competing interests.

## Authors' contributions

MJ and PH conceived and designed the experiments. LZ, JS and LX performed the experiments. MJ and LZ collected the samples and analyzed the data. BS and PH contributed reagents/materials/analysis tools. MJ and PH wrote the paper. All authors are in agreement with the content of the manuscript and this submission.
